# The incidence of coronary in-stent restenosis and the rate of reaching the standard of low-density lipoprotein cholesterol in patients with type 2 diabetes mellitus and unstable angina pectoris treated with ezetimibe and rosuvastatin

**DOI:** 10.3389/fcvm.2025.1599313

**Published:** 2025-09-08

**Authors:** Fanhao Ye, Hao Chen, Hebo Li

**Affiliations:** Department of Cardiology, Wenzhou People’s Hospital, The Wenzhou Third Clinical Institute Affiliated to Wenzhou Medical University, Wenzhou, Zhejiang, China

**Keywords:** T2DM, unstable angina, in-stent restenosis (ISR), ezetimibe, rosuvastatin

## Abstract

**Background:**

Diabetes is closely associated with the occurrence and development of coronary atherosclerotic heart disease. Coronary atherosclerosis is often severe and diffuse in patients with diabetes. We investigated the incidence of coronary in-stent restenosis (ISR) and the rate of reaching the standard of low-density lipoprotein cholesterol (LDL-C) in patients with type 2 diabetes mellitus (T2DM) and unstable angina pectoris (UAP) treated with ezetimibe and rosuvastatin one year later.

**Materials and methods:**

We selected the first pair of UAP patients with T2DM who underwent coronary artery stent implantation at our hospital between October 2018 and February 2022. According to drug use, the patients were divided into the rosuvastatin group [61 cases, rosuvastatin 10 mg/qn (every night)] and the combined group [60 cases, ezetimibe 10 mg/qd (once daily) and rosuvastatin 10 mg/qn]. Biochemical indices, left ventricular ejection fraction, and left ventricular end-diastolic diameter were collected before and one year after the first percutaneous coronary intervention. We collected data on the incidence of ISR and the rate of reaching the standard of LDL-C one year after surgery. Emergency PCI or coronary artery bypass grafting, cardiac death, and non-fatal acute myocardial infarction due to unstable angina pectoris 30 days after coronary stent implantation and lipid-lowering treatment were regarded as the primary endpoints.

**Results:**

After one year of follow-up, the incidence of in-stent restenosis(ISR), total cholesterol(TC), and LDL-C levels in the combined group[ISR, 3.33%; TC, 3.19 ± 0.75; LDL-C, 1.38(1.18–1.64)] were lower than those in the rosuvastatin group[ISR, 16.39% TC,C 3.84 ± 1.15; LDL-C, 1.92(1.52–2.61)] (*P* < 0.05). The rate of reaching the standard of LDL-C in the combined group (65%, 95% CI 0.560–0.809) was higher than that in the rosuvastatin group(31%, 95% CI 0.210–0.446) (*P* < 0.05). No significant difference in safety was observed between the two groups (*P* > 0.05). No endpoints were observed in the combined group.

**Conclusion:**

Resuvastatin combined with ezetimibe can better prevent ISR and reduce the incidence of cardiovascular adverse events. In addition, ezetimibe combined with rosuvastatin better reduced LDL-C levels.

## Background

Percutaneous coronary intervention (PCI) has ushered in a new era of treatment for coronary artery disease. However, in-stent restenosis (ISR) remains a potential post-PCI complication (1, 2). Despite the fact that drug-eluting stent (DES) implantation significantly lowers the clinical incidence of ISR compared to bare-metal stents, ISR still occurs in 3% to 20% of cases, primarily influenced by patient pathological characteristics, risk factors (especially diabetes), and the type of DES used (3, 4).

The incidence and prevalence of type 2 diabetes mellitus (T2DM) have surged in both developed and developing countries (5). There is a strong correlation between the development of cardiovascular disease and abnormal glucose metabolism (6). Among individuals with diabetes, the prevalence of coronary heart disease can reach as high as 55% (7).

The 2019 ESC and EAC guidelines for blood lipid management recommend that patients with T2DM at very high risk should aim to reduce their low-density lipoprotein cholesterol (LDL-C) levels by 50% from baseline and maintain LDL-C levels below 1.4 mmol/L (55 mg/dl) (8).

The primary objective of this study was to assess the incidence of ISR and the rate of achieving LDL-C targets in patients with T2DM and unstable angina pectoris (UAP) one year after treatment with ezetimibe and rosuvastatin.

## Materials and methods

### Study population

Patients with UAP and T2DM who underwent coronary stent implantation at Wenzhou People's Hospital between October 2018 and February 2022 were enrolled in the study. The inclusion criteria were as follows: (1) meeting the diagnostic criteria for T2DM and UA and (2) not having received statins or any other lipid-modulating drugs in the past 15 days. The exclusion criteria were as follows: (1) allergy to statins or ezetimibe; (2) active liver disease or liver dysfunction (alanine aminotransferase [ALT] level >1.5 times the upper limit of normal [ULN), as statins can exacerbate liver damage; (3) hypothyroidism, as statins may increase the risk of rhabdomyolysis in this condition; (4) history of alcohol or drug abuse, which can lead to liver damage; (5) homozygous familial hypercholesterolemia or familial dyslipoproteinemia, due to extremely high LDL-C levels that may be refractory to treatment, potentially biasing results; (6) myalgia or myasthenia of unknown cause, or creatine kinase (CK) level >1.5 times ULN, as atorvastatin can exacerbate muscle damage; (7) rheumatic immunologic diseases or tumors, as treatments for these conditions can affect blood lipids and bias results; and (8) resistance to aspirin and clopidogrel, as determined by platelet aggregation function tests (AA and ADP).

Based on the treatment regimen, the patients were divided into two groups: the rosuvastatin group (*n* = 61), who received rosuvastatin 10 mg/qn (every night), and the combination group (*n* = 60), who received rosuvastatin 10 mg/qn combined with ezetimibe 10 mg/qd (once daily). Both groups took their medications regularly for one year and received standard therapy, including aspirin, clopidogrel, nitrates, angiotensin-converting enzyme inhibitors or angiotensin receptor blockers, β-blockers, and oral hypoglycemic drugs.

### Anthropometric measurements and biochemical tests

Total cholesterol (TC), triglyceride (TG), high-density lipoprotein cholesterol (HDL-C), and LDL-C levels were assessed before PCI and one year after the first coronary stent implantation. Hemoglobin A1c (HbA1c), fasting blood glucose (FBG), ALT, creatinine (Cr), and CK were also measured. Left ventricular ejection fraction (LVEF) and left ventricular end-diastolic dimension (LVDD) were recorded using transthoracic echocardiography. One year post-treatment, coronary angiography was repeated to evaluate the stent stenosis. The criterion for achieving LDL-C targets after one year of treatment was based on the 2019 ESC and EAC blood lipid management guidelines, which stipulate that LDL-C levels should be <1.4 mmol/L or reduced by >50% from baseline.

The rate of achieving LDL-C targets was calculated as follows: (number of patients meeting the criteria/total number of patients) × 100%. ISR was defined as recurrent stenosis with a stent segment diameter >50% of the intravascular diameter. Adverse reactions were monitored throughout the treatment period, and the study was terminated if ALT exceeded 3 times ULN or CK levels exceeded three or five times the ULN, respectively.

### Endpoint event

**Figure 1:**
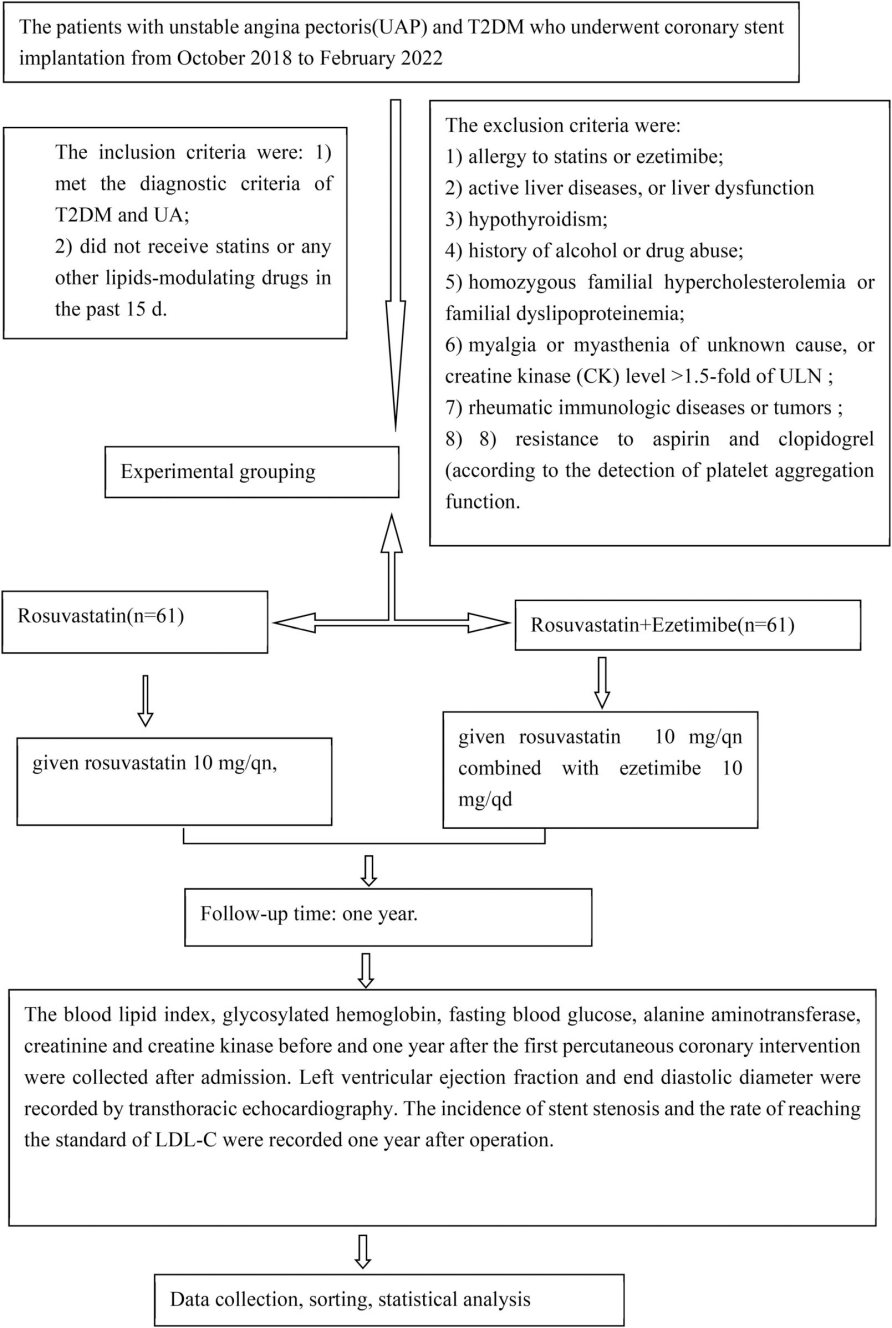
The study enrollment flow chart.

The primary endpoints included emergency percutaneous coronary intervention (PCI) or coronary artery bypass grafting (CABG), cardiac death, and non-fatal acute myocardial infarction attributable to unstable angina pectoris occurring within 30 days of coronary stent implantation during lipid-lowering therapy. The study enrollment flow chart is shown in Figure 1.

### Ethics approval and consent to participate

This study complied with the Declaration of Helsinki and was approved by the Medical Ethics Committee of Wenzhou People's Hospital. Written informed consent was obtained from all participants prior to their enrollment in the study (clinical trial number: 2018196).

### Statistical analysis

Statistical analyses were performed using SPSS version 23.0 for Windows (IBM Corp.; Armonk, NY). Categorical variables are presented as frequencies and percentages. Normally distributed continuous variables were expressed as mean ± standard deviation and analyzed using Student's *t*-test, while non-normally distributed data were reported as median (interquartile range) and analyzed using the Mann–Whitney *U-*test. Comparisons of categorical variables were conducted using either the chi-squared test or Fisher's exact test, with a *P*-value <0.05 considered statistically significant.

## Results

### Comparison of preoperative clinical characteristics between the two groups

No statistically significant differences were observed between the rosuvastatin group and the combination therapy group in terms of age, sex distribution, history of hypertension, severity of coronary artery disease, HbA1c levels, fasting blood glucose (FBG), alanine aminotransferase (ALT), creatinine (Cr), total cholesterol (TC), triglycerides (TG), high-density lipoprotein cholesterol (HDL-C), low-density lipoprotein cholesterol (LDL-C), non-HDL-C levels, or preoperative left ventricular end-diastolic diameter (LVDD) and left ventricular ejection fraction (LVEF) (all *P* > 0.05). The clinical characteristics of the patients in the two groups are presented in Table 1.

**Table 1:** Comparison of clinical characteristics in two groups before PCI.

Characteristics	Rosuvastatin (*n* = 61)	Rosuvastatin + Ezetimibe (*n* = 60)	*P* value
Age (yeat)	67.53 ± 9.88	64.20 ± 11.05	0.597
Male (%)	33 (54%)	39 (65%)	0.222
Hypertension (%)	50 (82%)	48 (80%)	0.783
Severity of myocardial infarction (%)			
Single vessel lesion (%)	18 (29.5%)	12 (20%)	0.226
Double vessel lesion, *n* (%)	11 (18%)	13 (21.7%)	0.616
Three vessel lesion, *n* (%)	32 (52.5%)	35 (58.3%)	0.516
HbA1c (%), mean ± SD	8.15 (6.90–9.43)	8.00 (7.10–8.70)	0.565
FBG (mmol/L), mean ± SD	7.91 (6.10–9.63)	7.07 (5.68–9.07)	0.158
ALT (U/L), mean ± SD	21 (15–32)	20 (15–28)	0.870
CK (U/L), mean ± SD	113.09 ± 70.86	110.65 ± 70.56	0.902
Cr (umol/L), mean ± SD	59.00 (48.00–77.50)	69 (57.25–80.00)	0.268
TC (mmol/L), mean ± SD	4.66 ± 0.93	4.68 ± 1.05	0.896
TG (mmol/L), mean ± SD	1.67 (1.00–2.45)	1.83 (1.26–2.71)	0.247
HDL-C (mmol/L), mean ± SD	1.02 (0.88–1.28)	0.95 (0.81–1.08)	0.054
LDL-C (mmol/L), mean ± SD	2.79 ± 0.92	2.77 ± 0.77	0.920
non-HDL-C (mmol/L), mean ± SD	3.56 ± 0.98	3.65 ± 1.06	0.621
LVEF (%), mean ± SD	65 (58.50–68.00)	65 (58.00–68.00)	0.629
LVDD (mm), mean ± SD	47 (43–50)	48(44–50)	0.805

HbA1c, hemoglobin A1c; FBG, fasting blood glucose; ALT, alanine aminotransferase; Cr, creatinine; TC, total cholesterol; TG, triacylglycerol; HDL-C, high-density lipoprotein cholesterol; LDL-C, low density lipoprotein-cholestero; non-HDL-C, none high-density lipoprotein cholesterol; LVEF, left ventricular ejection fraction; LVDD, left ventricular end diastolic dimension.

Comparison of biochemical indexes and in stent restenosis results between the two groups one year after operation.

There was no significant difference in baseline data such as HbA1c, FBG, ALT, Cr, TG, HDL-C, preoperative LVDD and preoperative LVEF between rosuvastatin group and combined group (*P* > 0.05). After one year of follow-up, the incidence of ISR, TC and LDL-C levels in the combined group[ISR 3.33%,TC 3.19 ± 0.75, LDL-C 1.38(1.18–1.64)] were lower than those in the rosuvastatin group[ISR 16.39%, TC 3.84 ± 1.15, LDL-C 1.92(1.52–2.61)]. The rate of reaching the standard of LDL-C in the combined group(65%, 95% CI 0.560–0.809) was higher than that in the rosuvastatin group(31%, 95% CI 0.210–0.446) (*P* < 0.05). The clinical characteristics of the patients in the two groups are shown in Table 2.

**Table 2:** Comparison of clinical characteristics in two groups one year after operation.

Characteristics	Rosuvastatin (*n* = 61)	Rosuvastatin + Ezetimibe (*n* = 60)	*P* value
HbA1c (%)	7.50 (6.90–8.30)	7.45 (6.63–8.28)	0.405
FBG (mmol/L)	7.23 (5.84–9.19)	6.80 (5.82–7.63)	0.075
ALT (U/L)	20 (12.5–29)	22 (16.25–28.75)	0.195
CK (U/L)	109.28 ± 71.10	112. ± 71.23	0.921
Cr (umol/L)	65 (52.5–78.5)	71 (57.25–83.00)	0.091
TC (mmol/L)	3.84 ± 1.15	3.19 ± 0.75	0.000
TG (mmol/L)	1.3 (0.98–1.66)	1.28 (0.99–1.75)	1.000
HDL-C (mmol/L)	0.98 (0.84–1.16)	0.98 (0.81–1.24)	0.758
LDL-C (mmol/L)	1.92 (1.52–2.61)	1.38 (1.18–1.64)	0.000
non-HDL-C (mmol/L)	2.70 (1.99–3.56)	1.97 (1.72–2.42)	0.000
LVEF (%)	65 (57–70)	65 (60–68.75)	0.840
LVDD (mm)	47 (44–50)	47.5 (44–49.75)	0.500
ISR (%)	10 (16.39%)	2 (3.33%)	0.016
The rate of reaching the standard of LDL-C (%)	19 (31%, 95% CI 0.210–0.446)	39 (65%, 95% CI 0.560–0.809)	0.000

HbA1c, hemoglobin A1c; FBG, fasting blood glucose; ALT, alanine aminotransferase; Cr, creatinine; TC, total cholesterol; TG, triacylglycerol; HDL-C, high-density lipoprotein cholesterol; LDL-C, low density lipoprotein-cholestero; non-HDL-C, none high-density lipoprotein cholesterol; LVEF, left ventricular ejection fraction; LVDD, left ventricular end diastolic dimension; ISR In stent restenosis.

### Comparison of adverse events

After one year of follow-up, no lipid-lowering drugs were stopped in either group due to impaired liver function, elevated creatine kinase levels, or myalgia. There were two endpoints in the rosuvastatin group (emergency PCI due to ACS). There were no end points in the combined group.

## Discussion

This study evaluated the effects of ezetimibe combined with rosuvastatin on the incidence of ISR and the LDL-C target achievement rate in patients with T2DM and UAP at a one-year follow-up. The results demonstrated that the combination therapy group exhibited a significantly lower incidence of ISR (at one year post-procedure) than the intermediate-intensity statin monotherapy group. Furthermore, the rosuvastatin-ezetimibe combination achieved lipid-lowering targets more rapidly than rosuvastatin alone, while maintaining an excellent safety profile. The absence of a high-intensity statin arm limits direct comparison of additive vs. dose-escalation strategies. However, our design reflects regional prescribing patterns where combination therapy increasingly supplements moderate statin doses.

ISR is defined as the progressive narrowing of the treated coronary artery segment due to arterial injury and subsequent neointimal hyperplasia after stent implantation. Typically occurring 6–12 months after PCI, ISR most commonly presents as recurrent angina pectoris but may also manifest as a myocardial infarction. As one of the primary factors compromising the long-term efficacy of PCI, ISR currently lacks standardized treatment strategies (9).

Recent advancements in coronary stent technology and antiplatelet therapies have reduced ISR incidence from historical rates of 40%–50% to approximately 10% (2). However, ISR prevention and management remain significant challenges in the field of cardiovascular medicine. The pathogenesis of ISR involves complex pathological mechanisms with multiple contributing factors, including: Current understanding suggests that various risk factors collectively induce endothelial cell injury or dysfunction, triggering local thrombosis and inflammatory responses in the body. This process, mediated by numerous cytokines, leads to smooth muscle cell proliferation, excessive extracellular matrix production, and vascular wall deposition, ultimately resulting in intimal hyperplasia and ISR (10). Notably, in-stent neoatherosclerosis may accelerate coronary plaque formation, promoting ISR progression.

Diabetes mellitus is an independent risk factor for ISR. Endothelial dysfunction mediated by advanced glycation end products plays a crucial role in ISR pathogenesis (11). These glycation products participate in post-PCI vascular remodeling processes, indicating that glycemic control alone cannot reverse the progression of vascular complications (12). Strict post-PCI glucose management combined with targeted AGE therapy may reduce ISR incidence (13, 14).

Lipid metabolism parameters demonstrate strong associations with ISR risk, with LDL-C being the most extensively studied and primary therapeutic target for dyslipidemia management (15). Substantial evidence indicates a positive correlation between serum LDL-C levels and ISR incidence, and LDL-C reduction effectively suppresses neointimal hyperplasia and delays ISR progression (16).

Abnormal lipid metabolism is the main cause of arteriosclerosis and a risk factor for coronary heart disease. Hyperlipidemia in patients leads to a large amount of lipid deposition on the vascular endothelium, resulting in endothelial cell proliferation and calcification, which results in gradual narrowing or even occlusion of the lumen. Abnormal blood lipid levels can be reflected by increased TG, TC, and LDL-C levels and/or decreased HDL-C levels. Therefore, all blood lipid regulation guidelines stipulate that the main purpose of reducing blood lipids is to reduce LDL-C and TC levels. Ezetimibe is a highly selective drug that interferes with cholesterol absorption. It can inhibit the protein transport of cholesterol in the small intestine, thus inhibiting the function of cholesterol uptake in the intestine, regulating the level of free cholesterol in the plasma, and reducing cholesterol storage in the liver. This product can be combined with statins to reduce LDL-C levels. While controlling the high-fat diet, it can be used independently to regulate blood lipids or in combination with HMG-CoA reductase inhibitors (statins) to treat hypercholesterolemia caused by various reasons, and it can significantly reduce the levels of LDL-C, TC, and apolipoprotein B in plasma. Ezetimibe regulates cholesterol by inhibiting its intestinal reabsorption; therefore, it is a new type of anti-lipid drug compared with statins. The pharmacological mechanism of this drug is to interfere with the transport of cholesterol by the NPC1L1 protein in the brush edge of the small intestine villus, so that the small intestine cannot fully absorb a large amount of cholesterol. It can also prevent the liver from storing and transporting cholesterol, regulate the level of cholesterol in the liver, increase the number of LDL-C receptors, and accelerate the metabolism of cholesterol in the plasma (17). The combination of ezetimibe and statins can play a synergistic role in preventing the generation of blood lipids from endogenous and exogenous sources, significantly reducing the level of cholesterol in plasma, reducing blood lipids, and slowing down the trend of atherosclerosis. These findings are in line with those of the CONNECT trial (18), showing that neoatherosclerosis was lower over a 3-year follow-up in patients undergoing intensive lipid-lowering therapy.

In our study, the sample size was small, and the follow-up period was one year. Therefore, more trials and long-term studies are needed to assess the clinical efficacy of rosuvastatin combined with ezetimibe in the treatment of patients with UAP complicated with T2DM after the first coronary stent implantation. While the observed ISR reduction was statistically significant, its magnitude should be interpreted cautiously given the small absolute event numbers. Confirmatory studies with larger cohorts are warranted.

## Conclusions

Compared with rosuvastatin alone, the combined use of ezetimibe can better reduce LDL-C levels, prevent in-stent restenosis, and reduce coronary artery disease and adverse events. Rosuvastatin combined with ezetimibe is safe.

## Data Availability

The original contributions presented in the study are included in the article/Supplementary Material, further inquiries can be directed to the corresponding author.
